# A low-cost recombinant glycoconjugate vaccine confers immunogenicity and protection against enterotoxigenic *Escherichia coli* infections in mice

**DOI:** 10.3389/fmolb.2023.1085887

**Published:** 2023-03-02

**Authors:** Asher J. Williams, Katherine F. Warfel, Primit Desai, Jie Li, Jen-Jie Lee, Derek A. Wong, Phuong M. Nguyen, Yufan Qin, Sarah E. Sobol, Michael C. Jewett, Yung-Fu Chang, Matthew P. DeLisa

**Affiliations:** ^1^ Robert F. Smith School of Chemical and Biomolecular Engineering, Cornell University, Ithaca, NY, United States; ^2^ Department of Chemical and Biological Engineering, Northwestern University, Technological Institute, Evanston, IL, United States; ^3^ Chemistry of Life Processes Institute, Northwestern University, Evanston, IL, United States; ^4^ Center for Synthetic Biology, Northwestern University, Technological Institute, Evanston, IL, United States; ^5^ Biochemistry, Molecular and Cell Biology, Cornell University, Ithaca, NY, United States; ^6^ Department of Population Medicine and Diagnostic Sciences, College of Veterinary Medicine, Cornell University, Ithaca, NY, United States; ^7^ Department of Bioengineering, Stanford University, Stanford, CA, United States; ^8^ Cornell Institute of Biotechnology, Cornell University, Ithaca, NY, United States

**Keywords:** synthetic glycobiology, cell-free (CF) protein synthesis, bacterial protein expression, conjugate vaccine, protein glycan coupling technology, protein glycosylation, oligosaccharyl transferase, vaccine carrier protein

## Abstract

Enterotoxigenic *Escherichia coli* (ETEC) is the primary etiologic agent of traveler’s diarrhea and a major cause of diarrheal disease and death worldwide, especially in infants and young children. Despite significant efforts over the past several decades, an affordable vaccine that appreciably decreases mortality and morbidity associated with ETEC infection among children under the age of 5 years remains an unmet aspirational goal. Here, we describe robust, cost-effective biosynthetic routes that leverage glycoengineered strains of non-pathogenic *E. coli* or their cell-free extracts for producing conjugate vaccine candidates against two of the most prevalent O serogroups of ETEC, O148 and O78. Specifically, we demonstrate site-specific installation of O-antigen polysaccharides (O-PS) corresponding to these serogroups onto licensed carrier proteins using the oligosaccharyltransferase PglB from *Campylobacter jejuni.* The resulting conjugates stimulate strong O-PS-specific humoral responses in mice and elicit IgG antibodies that possess bactericidal activity against the cognate pathogens. We also show that one of the prototype conjugates decorated with serogroup O148 O-PS reduces ETEC colonization in mice, providing evidence of vaccine-induced mucosal protection. We anticipate that our bacterial cell-based and cell-free platforms will enable creation of multivalent formulations with the potential for broad ETEC serogroup protection and increased access through low-cost biomanufacturing.

## Introduction

Enterotoxigenic *Escherichia coli* (ETEC) is a primary cause of diarrheal disease worldwide and the leading cause of traveler’s diarrhea, especially in locations where clean water and sanitation remain limited (Qadri et al., 2005; Khalil et al., 2018). In addition to acute diarrhea-associated morbidity, ETEC is also a leading cause of mortality, disproportionately affecting children under the age of 5 years who lack immunity from prior exposure (Kotloff et al., 2013; Lamberti et al., 2014). While deaths from infectious diarrhea have been declining for several decades, due in part to oral rehydration therapy, ETEC continues to wreak havoc in terms of acute morbidity and associated sequelae such as growth stunting, malnutrition, and cognitive impairment (Guerrant et al., 2013; Kotloff et al., 2017; Fleckenstein and Kuhlmann, 2019).

A vaccine that prevents both acute illness as well as the sequelae associated with ETEC infection has long been a priority of the World Health Organization (WHO) (WHO 2020 WHO Product Development for Vaccines Advisory Committee (PDVAC), 2020). At present, vaccine development is primarily centered on a subset of important virulence factors including colonization factor/cell surface (CF/CS) antigens and enterotoxins, namely, heat-stable toxin (ST) and heat-labile toxin (LT) (Bourgeois et al., 2016; Riddle et al., 2018; Fleckenstein, 2021; Khalil et al., 2021). These factors comprise a classical model for ETEC molecular pathogenesis in which ETEC colonizes the small intestine using plasmid-encoded CF/CS antigens followed by production of one or more enterotoxin (*e.g.*, LT, ST) that drive fluid export and diarrhea (Fleckenstein et al., 2010). Despite significant efforts over several decades, human trials involving vaccine candidates based on this classical paradigm have met limited success (Darsley et al., 2012; Behrens et al., 2014; Harro et al., 2019; Khalil et al., 2021). Hence, further improvements are needed to increase the levels of protection against more severe ETEC diarrhea and to expand protection to the breadth of ETEC serotypes. Consequently, there are still no licensed vaccines for ETEC.

To expand the repertoire of antigens that could be targeted in future vaccine designs, a handful of studies have assessed adaptive immune responses in humans following either experimental challenge with ETEC or oral administration of an inactivated ETEC vaccine (Fleckenstein et al., 2014; Chakraborty et al., 2018a; Svennerholm et al., 2022). Several non-canonical antigens beyond the classic vaccine targets were identified, including secreted proteins (e.g*.*, EatA, EtpA, and YghJ), cell surface-expressed proteins (*e.g.*, Ag43, OmpW), and lipopolysaccharide (LPS), among others. Unlike the secreted and cell-surface protein antigens, LPS are glycolipids that include an outermost O-antigen polysaccharide (O-PS) component that is composed of repeating subunits that extend from the surface of the bacteria (Raetz et al., 2002). Interestingly, the inactivated ETEC vaccine strain, ETVAX, elicited response frequencies in infants against serogroup O78 LPS (which is expressed on the strain) that were comparable or higher than those elicited against the CFs present in the vaccine strain. This suggests that LPS is a potent antigen that may contribute to vaccine-induced protection (Svennerholm et al., 2022). Other studies have indicated that O-PS is common among ETEC strains that cause diarrheal illness (Wolf, 1997; Begum et al., 2014), with more than 78 O serogroups identified in ∼1,000 ETEC isolates from widespread locations (Wolf, 1997). While this number is impractically high for developing a broadly protective, multivalent vaccine, 10 of these serogroups (O6, O8, O9, O25, O27, O78, O128, O148, O153 and O159) account for >75% of the isolates, suggesting that a 10-valent polysaccharide vaccine could afford broad protection with the fewest components possible. At present, however, virtually no attention has been paid to ETEC LPS/O-PS as a subunit vaccine antigen.

One barrier to the development of an LPS/O-PS-based vaccines in general is the fact that purified polysaccharides, while moderately immunogenic in adults, are often entirely unable to provoke a humoral response in infants and children, the population in greatest need of an ETEC vaccine. This problem results from an inability of polysaccharides to interact with the receptors on T cells, but can be solved by covalently coupling the LPS or O-PS structure to an immunogenic protein carrier that serves as a CD4^+^ T cell-dependent antigen (Avery and Goebel, 1929). The resulting conjugates invoke a T-cell response that results in strong polysaccharide-specific antibody responses, immunological memory, and high immunogenicity in young children (Rappuoli, 2018). Indeed, conjugates are a safe and effective strategy for protecting against virulent pathogens, with successful vaccines licensed worldwide against *Haemophilus influenzae*, *Neisseria meningitidis* serogroups (tetravalent), *Streptococcus pneumoniae* (up to 20-valent), and *Salmonella typhi*, and other promising candidates in various stages of clinical development (Rappuoli, 2018).

Although effective, traditional conjugate vaccines have several drawbacks. Most notably is the complex, multistep process required to purify, isolate, and conjugate bacterial polysaccharides, which is costly, time and labor intensive, and low yielding (Frasch, 2009). To sidestep these issues, metabolic engineering of bacteria has emerged as an attractive alternative for one-step biosynthesis of an unlimited and renewable supply of conjugate vaccines (Kay et al., 2019). This bioconjugation approach leverages engineered protein glycosylation in non-pathogenic *E. coli* strains that are capable of conjugating recombinantly produced O-PS molecules to co-expressed carrier proteins by an oligosaccharyltransferase (OST) such as PglB from *Campylobacter jejuni* (*Cj*PglB) (Feldman et al., 2005). To date, several unique conjugates have been produced by this method, with a few currently under clinical investigation (Kay et al., 2019; ClinicalTrials.gov, 2021; ClinicalTrials.gov, 2022). Building on these cell-based efforts, we recently described a method called iVAX (*in vitro* conjugate vaccine expression) that enables conjugate vaccine biosynthesis using cell-free lysates derived from glycosylation-competent *E. coli* strains (Stark et al., 2021). The iVAX platform has the potential to shorten vaccine development timelines and enable distributed, cold chain–independent vaccine manufacturing by harnessing cell extracts to generate conjugates *in vitro*.

In the current study, we describe two biosynthetic routes—one cell-based and the other cell-free—for low-cost production of conjugate vaccine candidates against two of the most prevalent O serogroups of ETEC, O148 and O78 (Wolf, 1997). Both routes enabled site-specific installation of ETEC O-PS onto carrier proteins used in licensed vaccines, namely, the non-acylated form of protein D (PD) from *H. influenzae* and cross-reactive material 197 (CRM_197_), a genetically detoxified variant of the *Corynebacterium diphtheriae* toxin (DT). The resulting conjugates stimulated strong O-PS-specific IgG antibody titers in mice, with the resulting antibodies possessing bactericidal activity against the cognate pathogens. For one of the prototype conjugates decorated with serogroup O148 O-PS, we further demonstrated observed reduction of ETEC colonization in mice, suggesting some amount of mucosal protection. Overall, our work expands the inventory of antigens for ETEC vaccine design and provides an important first step towards the creation of a custom, multivalent vaccine with potential for broad ETEC coverage and increased access through adoption of simplified, low-cost biomanufacturing platforms.

## Results

### Expression of ETEC serogroup O148 O-PS antigen in non-pathogenic *Escherichia coli* cells

**FIGURE 1:**
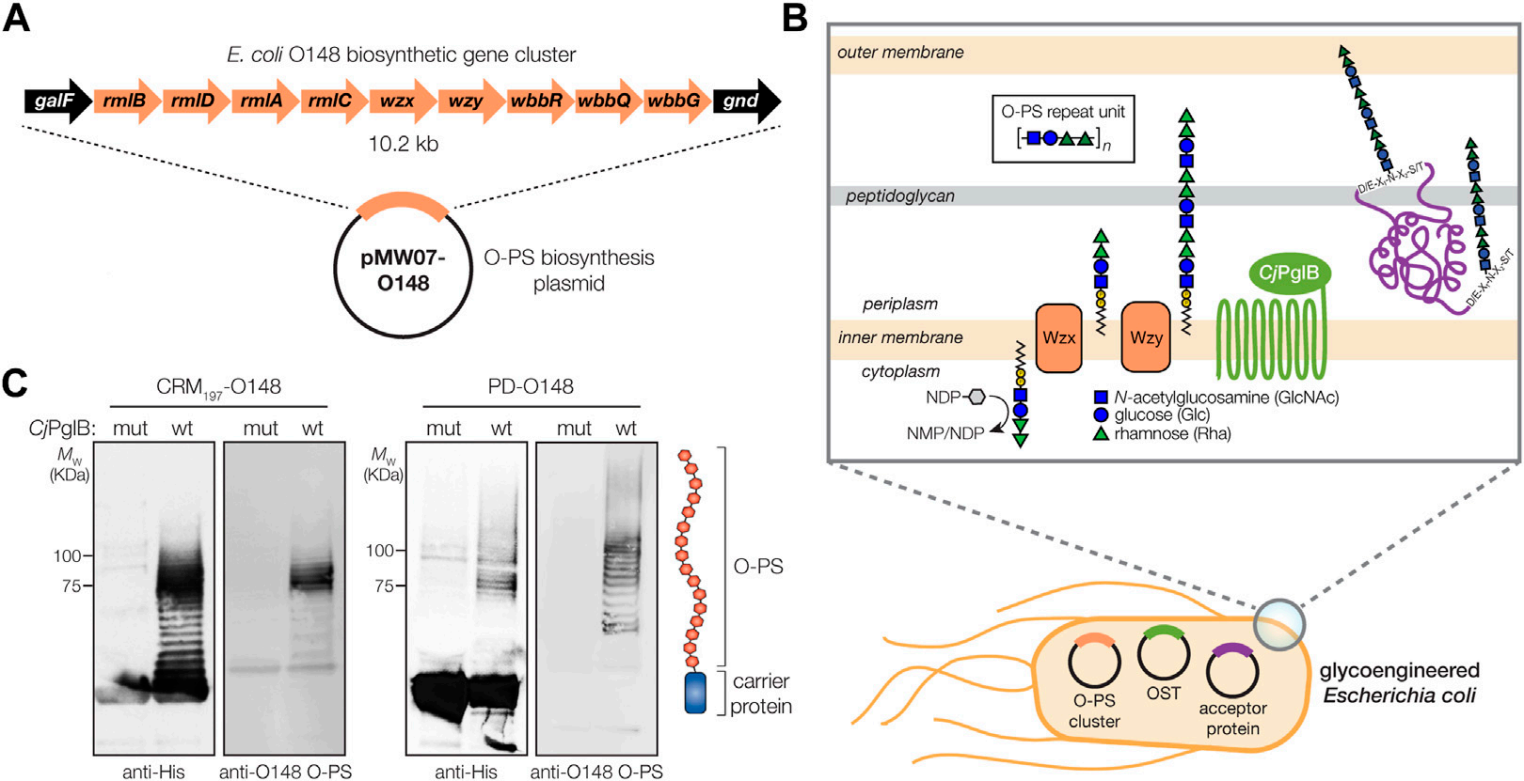
Biosynthesis of glycoconjugate vaccine candidates against ETEC bacteria. **(A)** Biosynthesis of ETEC O148 O-PS from plasmid pMW07-O148, which encodes the entire O-PS locus from ETEC strain B7A (serotype O148:H28) between *galF* and *gnd*. **(B)** Glycoconjugate biosynthesis is enabled by combining engineered O-antigen biosynthesis with *N*-linked protein glycosylation by *C. jejuni* PglB (*Cj*PglB). Several plasmid-encoded glycosyltransferases sequentially add the repeat-unit sugars to the lipid carrier undecaprenol pyrophosphate in the cytoplasmic membrane. The lipid-linked oligosaccharide is flipped by Wzx and polymerized by the Wzy polymerase, and subsequently transferred to “DQNAT” acceptor sites in the carrier protein by PglB. Deletion of *waaL* in the host strain is used to eliminate transfer of the O-PS to lipid A-core and ensure efficient transfer to the acceptor protein, while deletion of *lpxM* results in pentaacylated lipid A structure having significantly reduced toxicity. **(C)** Immunoblot analysis of purified carrier proteins derived from *Escherichia coli* CLM24 cells carrying a plasmid encoding either CRM_197_^4xDQNAT^ or PD^4xDQNAT^ along with plasmid pMW07-O148 encoding the ETEC O148 O-PS biosynthetic pathway and plasmid pMAF10 encoding wild-type *Cj*PglB (wt) or an inactive mutant of *Cj*PglB (mut) as indicated. Blots were probed with anti-His antibody to detect acceptor proteins and anti-ETEC O148 antibody to detect O-PS. Images depict aglycosylated and multiply glycosylated forms of CRM_197_^4xDQNAT^ or PD^4xDQNAT^. Molecular weight (*M*_W_) markers are indicated on the left. Results are representative of three biological replicates.

Biosynthesis of the O-PS antigen from ETEC serogroup O148 involved plasmid pMW07-O148 (Celik et al., 2015; Chen et al., 2016), which encodes the 10.2 kb O-PS gene cluster from ETEC strain B7A (serotype O148:H28) (DuPont et al., 1971) (Figure 1A). To confirm O-PS expression, we took advantage of the fact that O-PS antigens assembled in the cytoplasmic membrane of *E. coli* cells are transferred onto lipid A-core by the O-antigen ligase, WaaL. The lipid A-core-linked O-PS molecules are then shuttled to the outer membrane, becoming displayed on the cell surface where they are readily detectable with antibodies or lectins having specificity for the O-PS structure. As expected, non-pathogenic *E. coli* W3110 cells, which carry a copy of the *waaL* gene, were observed to express the ETEC O-PS antigen on their surface as evidenced by cross reactivity of nitrocellulose-spotted cells with an anti-ETEC O148 antibody (Supplementary Figure S1A). The binding observed for these cells was on par with that measured for ETEC strain B7A (Levine et al., 1979), which natively expresses the O148 O-PS antigen. In contrast, both plasmid-free W3110 cells and W3110 cells carrying empty pMW07 plasmid showed little to no cross reactivity. A similar lack of cross reactivity was observed for CLM24 cells, which have a deletion in the *waaL* gene, confirming that the recombinant O-PS antigen was assembled *via* the canonical lipid A-core pathway.

### Glycosylation of licensed vaccine carrier proteins with ETEC O148 O-PS

To generate a strong IgG response and lasting immunity, it is desirable to use a highly immunogenic protein as a carrier for the polysaccharide antigen, in this case ETEC serogroup O148 O-PS. To this end, we sought to engineer non-pathogenic *E. coli* with the ability to glycosylate a set of carrier proteins, namely, PD and CRM_197_, that are currently used in licensed conjugate vaccines (Figure 1B). To enable conjugation of O-PS antigens to these carrier proteins, both were modified at their C termini with four tandem repeats of an optimized bacterial glycosylation motif, DQNAT (Chen et al., 2007), followed by a 6x-His tag to enable detection *via* Western blot analysis and purification by Ni-NTA chromatography. A signal peptide sequence derived from the *E. coli* DsbA protein was fused to the N-terminus to localize CRM_197_ and PD to the periplasm in a manner that is compatible with *N*-linked glycosylation (Fisher et al., 2011). Each of the resulting plasmids, pTrc99A-CRM_197_^4xDQNAT^ and pTrc99A-PD^4xDQNAT^, were used to transform *E. coli* strain CLM24 carrying plasmid pMW07-O148 that encoded the O-PS biosynthetic enzymes and plasmid pMAF10 that encoded *Cj*PglB (Feldman et al., 2005). CLM24 cells were used because they have a deletion of the gene encoding the WaaL O-antigen ligase that makes undecaprenol pyrophosphate (UndPP)-linked glycans including O-PS structures exclusively available to *Cj*PglB by preventing their unwanted transfer to lipid A-core (Feldman et al., 2005).

Following overnight expression of *Cj*PglB along with either CRM_197_^4xDQNAT^ or PD^4xDQNAT^ in the presence of the ETEC serogroup O148 biosynthetic enzymes, cells were lysed, and His-tagged carrier proteins were purified by Ni-NTA chromatography. Elution fractions from each sample were separated by SDS-PAGE and subjected to immunoblotting using an anti-His antibody to detect the carrier proteins or antiserum specific for ETEC O148 LPS to detect the O-PS antigen. This analysis revealed that both CRM_197_^4xDQNAT^ and PD^4xDQNAT^ were readily glycosylated with ETEC O148 O-PS glycans (Figure 1C). Importantly, we observed a ladder-like banding pattern for both O148 O-PS-linked carrier proteins (hereafter CRM_197_-O148 and PD-O148), which is characteristic of *Cj*PglB-mediated O-PS transfer (Feldman et al., 2005) and results from variability in the chain length of O-PS antigens generated by the Wzy polymerase (Raetz et al., 2002). The most intense laddering signal was observed above 75 kDa, suggesting that the carriers were heavily decorated with ETEC O148 O-PS structures comprised of >10 repeating units (RUs). Control reactions with CLM24 cells that lacked the O-PS biosynthetic plasmid or expressed a catalytically inactive *Cj*PglB enzyme, generated by introducing D54N and E316Q substitutions (Ollis et al., 2014), confirmed that O-PS conjugation to the carrier proteins depended on both the O-PS biosynthetic enzymes and *Cj*PglB (Figure 1C; shown for mutant *Cj*PglB).

### Immunogenicity of PD-O148 glycoconjugate in mice

**FIGURE 2:**
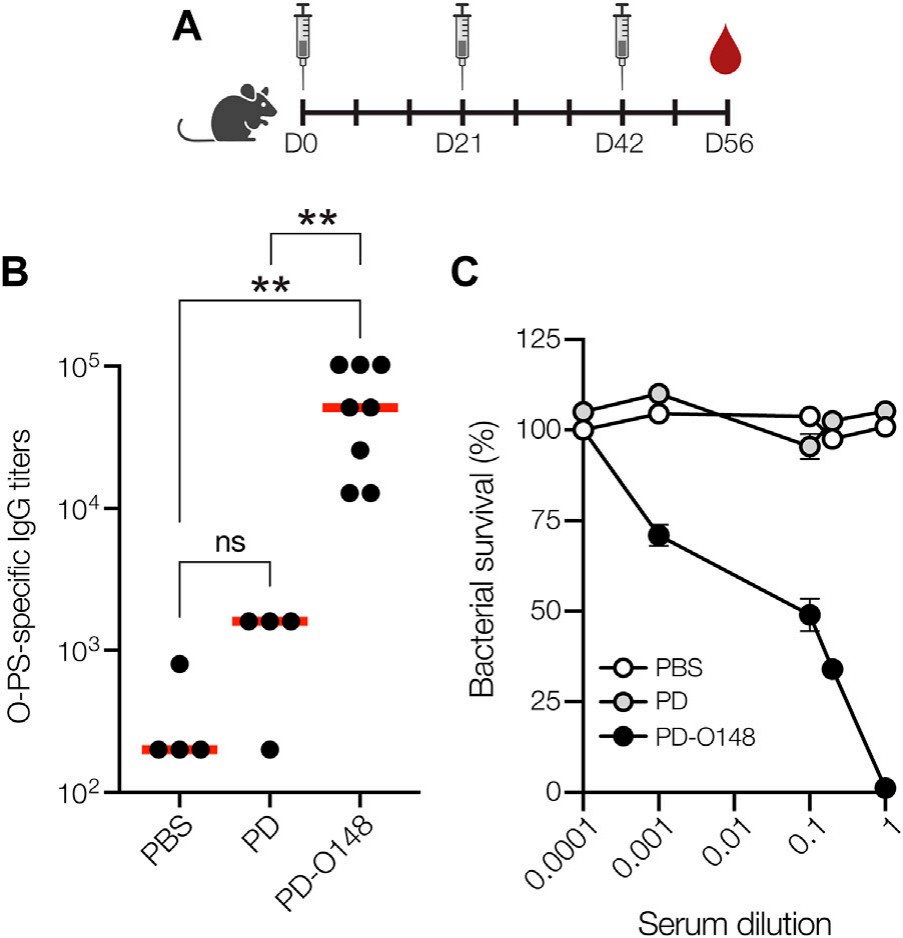
Glycoconjugates are immunogenic. **(A)** Schematic of the prime-boost immunization schedule. Mice received an initial injection on day 0 and identically formulated booster injections on days 21 and 42. Blood was drawn on days 0, 35, 49, and at study termination on day 56. **(B)** O-PS-specific IgG titers in day 56 serum of individual mice (black circles) and mean titers of each group (red lines) as determined by ELISA with LPS derived from ETEC strain B7A (serotype O148:H28) as immobilized antigen. Groups of eight BALB/c mice were immunized s. c. with 100 μL PBS alone or PBS containing 50 μg of either aglycosylated PD carrier protein or glycosylated ETEC O148 conjugate (PD-O148) adjuvanted with aluminium phosphate adjuvant. Mice were boosted on days 21 and 42 with identical doses of each immunogen. Data were analyzed for statistical significance using the Mann-Whitney test (*p < 0.05; **p < 0.01; ns, not significant). **(C)** Bactericidal killing activity of antibodies in the serum of mice immunized with PBS, unmodified PD carrier protein, or ETEC O148 conjugate (PD-O148). Survival data were derived from a standard SBA where dilutions of serum from immunized mice were tested against ETEC strain B7A (serotype O148:H28) in the presence of human complement. SBA curves were generated with pooled sera from each group. Data are the mean of three biological replicates ±SEM.

To investigate conjugate immunogenicity, BALB/c mice were immunized subcutaneously (s.c.) with glycosylated PD bearing the ETEC O148 O-PS antigen and serum from these animals was analyzed by enzyme-linked immunosorbent assay (ELISA) to determine antibody titers. BALB/c mice were immunized with 50 μg doses of protein, either PD alone or PD-O148 conjugate, adjuvanted with aluminium phosphate, and subsequently injected with identical booster doses at 21 and 42 days after the initial injection (Figure 2A). Upon analyzing sera collected on day 56, we found that BALB/c mice receiving the PD-O148 conjugate produced high titers of IgG antibodies that specifically recognized LPS derived from ETEC strain B7A (Figure 2B). These serum IgG levels were significantly elevated (∼2 orders of magnitude) compared to the titers measured in sera of control mice receiving PBS or aglycosylated PD. The ability of the PD-O148 conjugate to elicit strong IgG titers against ETEC B7A LPS provides further validation for using non-pathogenic *E. coli* strains engineered with protein glycosylation machinery as hosts for conjugate vaccine production.

### Neutralization of ETEC by vaccine-induced serum antibodies

Previous studies have shown that the elicitation of neutralizing serum antibodies against ETEC in mice can provide possible insight to vaccine efficacies (Yang et al., 2011). Therefore, PD-O148 conjugate vaccine-induced serum antibodies were evaluated for the ability to promote complement-mediated killing of ETEC strain B7A by serum bactericidal assay (SBA). SBA is an established method by which the activity of IgGs against bacterial pathogens can be measured. It often correlates with protection for serotypes of a pathogen and is a key *in vitro* method for measuring the bactericidal activity of antibodies (Borrow et al., 2005). Of relevance here, several groups have developed bactericidal assays for evaluating whether serum IgG antibodies can potentiate the killing of different ETEC strains (Yang et al., 2011; Sears et al., 2017). Using a similar methodology, we investigated whether O-PS-specific serum IgGs elicited by the PD-O148 glycoconjugate would recognize the corresponding LPS on the exterior of ETEC strain B7A and mediate bacteriolysis of the enteric pathogen in the presence of components of the human complement system. For the sera derived from mice vaccinated with the PD-O148 conjugate, ∼50% survival of ETEC B7A cells (corresponding to ∼50% killing activity) was observed at dilutions as high as 10-fold (Figure 2C). In contrast, virtually no killing was observed for sera derived from mice treated with PBS or the aglycosylated PD carrier protein as evidenced by pathogen survival that was close to 100%. Additionally, near complete killing was observed for the undiluted sera of vaccinated mice, whereas no killing was observed for the PBS and aglycosylated PD groups at the same serum dilution. These results confirm the bactericidal functionality of PD-O148 conjugate vaccine-induced IgGs present in the sera of immunized mice and predict the efficacy and protectiveness of our glycoengineered vaccine candidate.

### Protective efficacy of PD-O148 glycoconjugate in mice

**FIGURE 3:**
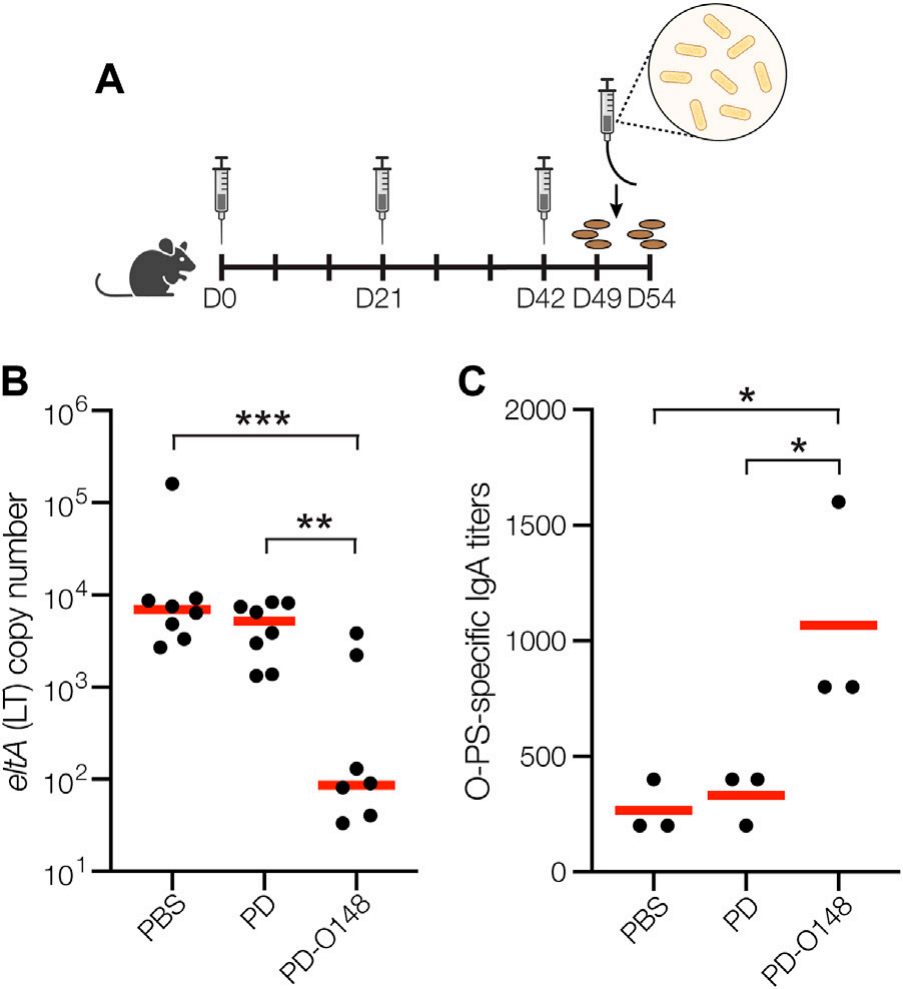
Glycoconjugates are functional and protective. **(A)** Schematic of the immunization and challenge schedule. Mice received an initial immunogen injection on day 0 and identically formulated booster injections on days 21 and 42. On day 49, mice received antibiotics in the drinking water to eradicate normal flora and fecal pellets were collected. On day 51, mice were subsequently infected by gavage and checked daily for 3 days. On day 54, fecal pellets were collected, and mice were sacrificed. **(B)** Vaccine effects on stool shedding of ETEC B7A in infected mice 3 days post-infection. Groups of eight BALB/c mice receiving PBS, unmodified PD carrier protein, or PD-O148 were infected with ∼1 × 10^4^ CFUs of ETEC B7A. Fecal samples were collected for DNA extraction and analyzed by qPCR to detect pathogen. ETEC burden in fecal pellets was measured by qPCR of the *eltA* gene, which encodes heat-labile endotoxin (LT). Data are the mean (red bars) of individual mice (black circles). **(C)** O-PS-specific SIgA titers in day 49 fecal pellet extracts pooled from pairs of mice within the same group (black circles) and mean titers of each group (red lines) as determined by ELISA with LPS derived from ETEC strain B7A (serotype O148:H28) as immobilized antigen. Data in **(B)** and **(C)** were analyzed for statistical significance using the Mann-Whitney test (*p < 0.05; **p < 0.01; ***p < 0.001; ns, not significant).

Encouraged by the immunogenicity of our PD-O148 conjugate and its ability to elicit bactericidal antibodies, we next tested the ability of the PD-O148 conjugate to protect mice in a murine model of an orally administered ETEC B7A infection. A major hurdle in developing enteric vaccines is the lack of a suitable small animal model to study the efficacy and immunogenicity of potential ETEC vaccines prior to testing in larger animals or humans. Nevertheless, ETEC infection is commonly induced by the pathogen *via* oral administration, with mice becoming colonized with ETEC following oral challenge using inocula as small as 10^3^ colony-forming units (CFUs) (Fleckenstein and Rasko, 2016). Oral gavage was selected as an infection model as it closely reflects the route of infection and potentially recapitulates relevant outcomes of ETEC infection seen in humans (Bolick et al., 2018). In this study, mice were pretreated with the antibiotic streptomycin, allowing them to more closely mimic the disease symptoms that are often seen in humans (Bolick et al., 2018; Medeiros et al., 2020). Following immunization according to an identical schedule as above, mice were infected by oral gavage with ∼1 × 10^4^ CFUs of ETEC strain B7A (Figure 3A). After challenge infection, mice were checked for signs and symptoms of enteric illness like watery diarrhea, and stool samples were also collected to detect shedding of the challenge strain. Diarrheal illness, when it occurs, is associated with higher fecal shedding levels of ETEC in animals and humans (Chakraborty et al., 2018b; Bolick et al., 2018). Post-challenge shedding levels of the challenge strain ETEC B7A was detected using quantitative PCR (qPCR) to examine fecal pellet DNA extracts for the presence of LT encoded by the *eltA* gene*.* Only mice receiving the PD-O148 conjugate exhibited a significant reduction in ETEC stool shedding detected at 3 days post-infection (∼2-log reduction compared to the PBS and aglycosylated PD carrier protein control groups) (Figure 3B), coinciding with no detectable watery diarrhea and confirming the ability of our conjugate vaccine to reduce colonization and promote clearance of the infection.

While it is possible that the neutralizing serum IgGs elicited by our conjugate may have contributed to this reduced ETEC shedding, it is well established that protective immunity against non-invasive intestinal pathogens such as ETEC depends on the induction of both a systemic immune response and a mucosal immune response involving production of secretory immunoglobulin A (SIgA) antibodies at mucosal surfaces (Levine et al., 1979). To determine whether our s. c. administered glycoconjugate elicited such a mucosal immune response, we measured O-PS-specific SIgA antibody titers in fecal pellet extracts collected from mice 48 h prior to challenge infection. Importantly, the PD-O148 conjugate elicited significantly elevated titers of O148 O-PS-specific SIgA antibodies that were ∼4-fold greater than the titers measured in sera of control mice receiving PBS or aglycosylated PD (Figure 3C). Taken together, these results demonstrate the ability of the PD-O148 conjugate to trigger both systemic and mucosal immune responses, which overall resulted in a significant reduction in ETEC colonization.

### Biosynthesis and immunogenicity of an ETEC serogroup O78-directed conjugate

As a first step towards such a multivalent formulation, we sought to create a glycoconjugate vaccine candidate against ETEC serogroup O78 by implementing an identical strategy as outlined above for serogroup O148. We chose serogroup O78 because it is one of the most widely distributed and most frequently occurring ETEC serogroups that, together with ETEC serogroups O6, O8, O27, O148, O153 and O169, accounts for nearly 25% of the nearly 1,000 isolated identified worldwide (Wolf, 1997). Introduction of plasmid pMW07-O78 (Chen et al., 2016; Stark et al., 2021; Warfel, 2022) (Supplementary Figure S2A) into non-pathogenic *E. coli* strain W3110 enabled expression of cell-surface O78 O-PS molecules that were readily detected by dot blot analysis (Supplementary Figure S1B). By combining plasmid pMW07-O78 together with the plasmids for expressing *Cj*PglB and the CRM_197_ carrier protein in CLM24 Δ*lpxM* cells, we were able to produce glycosylated CRM_197_ bearing the O78 O-PS antigen (Supplementary Figure S2B). The glycosylated CRM_197_-O78 conjugate was used to immunize BALB/c mice and was observed to induce high titers of IgG antibodies that specifically recognized LPS derived from ETEC strain H10407 (serotype O78:H11) (Levine et al., 1980) (Supplementary Figure S2C). Collectively, these results highlight the modularity of the biosynthetic approach, enabling facile production of an additional serogroup-specific conjugate simply by swapping out the O-PS biosynthesis plasmid. Such interchangeability will be key to generating a multivalent conjugate formulation that confers broad ETEC serogroup protection, with the results here representing an important first step in that direction.

### Cell-free biosynthesis of ETEC O78 O-PS conjugate

**FIGURE 4:**
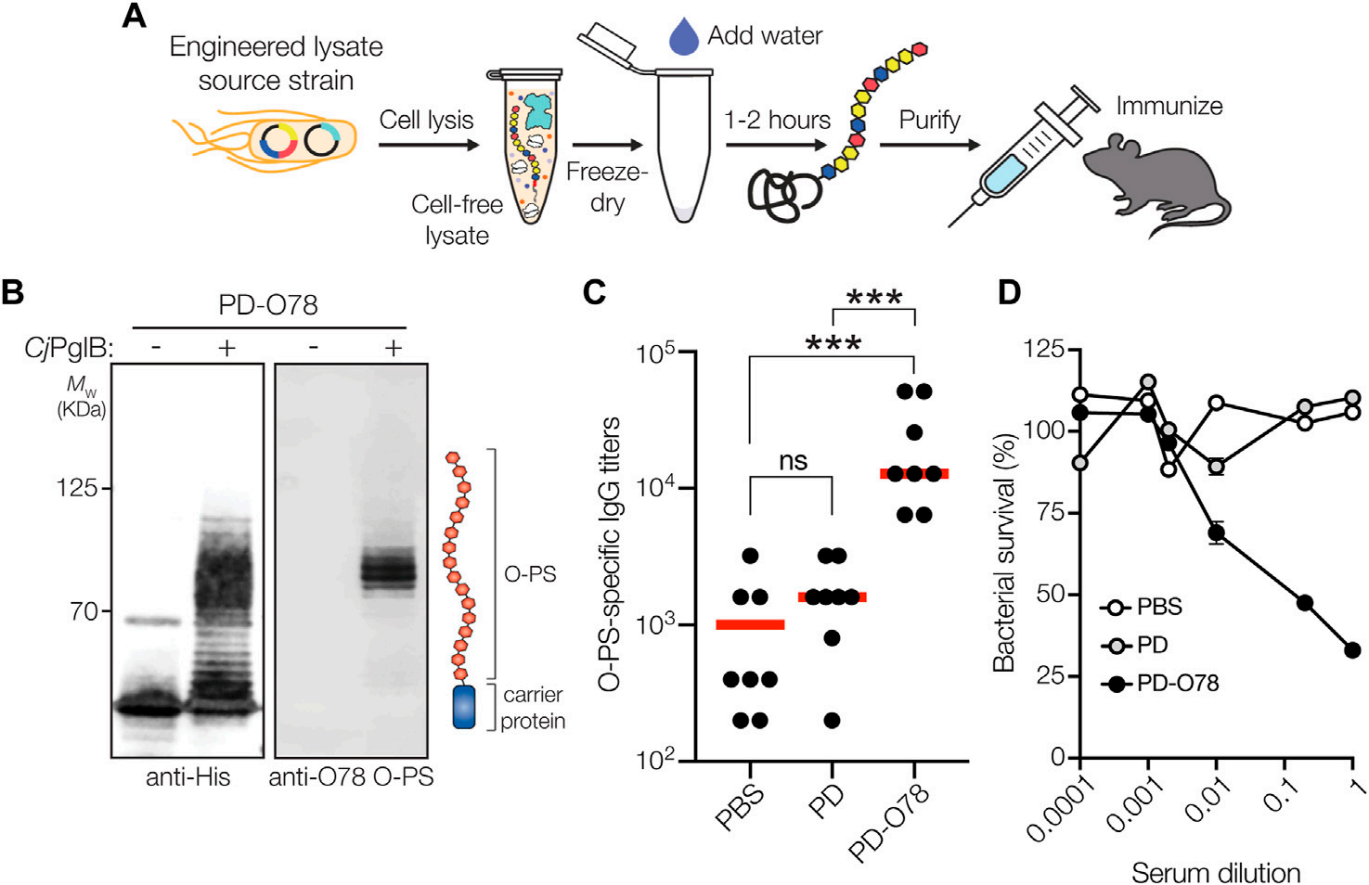
Glycoconjugates are functional and protective. **(A)** The iVAX platform provides a rapid means to develop and distribute conjugate vaccines against bacterial pathogens. **(B)** Immunoblot analysis of PD^4xDQNAT^ generated using iVAX lysate. Specifically, 5-mL reactions with glyco-enriched S12 extract derived from CLM24 Δ*lpxM* cells carrying pMW07-O78 and pSF-*Cj*PglB-LpxE were primed with plasmid pJL1-PD^4xDQNAT^. Blots were probed with anti-His antibody to detect acceptor proteins and anti-ETEC O78 antibody to detect O-PS antigens. Images depict aglycosylated and multiply glycosylated forms of PD^4xDQNAT^. Molecular weight (*M*_W_) markers are indicated on the left. Results are representative of three biological replicates. **(C)** O-PS-specific IgG titers in day 56 serum of individual mice (black circles) and mean titers of each group (red lines) as determined by ELISA with LPS derived from ETEC strain H10407 (serotype O78:H11) as immobilized antigen. Groups of eight BALB/c mice were immunized s. c. with 100 μL PBS alone or PBS containing 24 μg of either aglycosylated PD carrier protein or glycosylated ETEC O78 conjugate adjuvanted with aluminium phosphate adjuvant. Mice were boosted on days 21 and 42 with identical doses of each immunogen. Data were analyzed for statistical significance using the Mann-Whitney test (*p < 0.05; **p < 0.01; ***p < 0.001; ns, not significant). **(D)** Bactericidal killing activity of serum antibodies from mice immunized with same immunogens as in **(C)**. Survival data were derived from a standard SBA where serum dilutions were tested against ETEC strain H10407 in the presence of human complement. SBA curves were generated with pooled sera from each group. Data are the mean of three biological replicates ±SEM.

In parallel to using living cells, we also explored whether a cell-free protein synthesis (CFPS) approach could be used to produce glycoengineered conjugate vaccine candidates against ETEC. To this end, we leveraged iVAX technology (Stark et al., 2021) (Figure 4A) that had previously been used to synthesize clinically relevant doses of protective conjugate vaccines comprised of pathogen-specific O-PS antigens linked to licensed carrier proteins. Here, we produced an iVAX lysate from *E. coli* CLM24 Δ*lpxM* cells expressing the ETEC O78 O-PS biosynthetic pathway and *Cj*PglB. This lysate, which contained lipid-linked ETEC O78 O-PS and active *Cj*PglB, was used to catalyze iVAX reactions primed with plasmid DNA encoding the PD^4xDQNAT^ carrier protein. The products of these reactions were immunoblotted with anti-His antibody or a commercial anti-ETEC O78 antibody specific to the ETEC O78 O-PS. Similar to cell-based expression, cell-free iVAX reactions produced PD^4xDQNAT^ that was clearly glycosylated with the O78 O-PS antigen and exhibited the characteristic ladderlike banding pattern associated with O-PS chain-length variability (Figure 4B). Control reactions with lysates from cells lacking the ETEC O78 O-PS were devoid of any detectable glycosylation. Following immunization of BALB/c mice with the iVAX-derived conjugate, we observed strong induction of IgG antibodies that specifically recognized LPS derived from ETEC strain H10407 (serotype O78:H11) (Figure 4C), with serum IgG titers comparing favorably to those observed following immunization with the CRM_197_-O78 conjugate that was made in living cells. Finally, PD-O78 conjugate vaccine-induced serum antibodies were evaluated for the ability to promote complement-mediated killing of ETEC strain H10407 by SBA. Greater than ∼50% killing activity of ETEC H10407 cells was observed for the sera derived from PD-O78-vaccinated-mice at dilutions as high as 10-fold, whereas no measurable killing was observed for sera derived from mice treated with PBS or aglycosylated PD (Figure 4D).

## Discussion

In the present study, we describe robust cell-based and cell-free bioconjugation strategies for producing conjugate vaccine candidates against two widespread O serogroups of ETEC, O148 and O78 (Wolf, 1997). These strategies leveraged glycoengineered strains of non-pathogenic *E. coli* and their cell-free extracts for site-specific installation of two different ETEC O-PS structures onto the PD and CRM_197_ carrier proteins that are used in licensed vaccines. The resulting PD- and CRM_197_-based conjugates were strongly immunogenic in mice, eliciting high titers of O-PS-specific IgG antibodies against ETEC strains B7A (serotype O148:H28) and H10407 (serotype O78:H11).

In addition to induction of a systemic immune response, the PD-O148 conjugate also triggered a mucosal immune response as evidenced by the elevated fecal SIgA antibody titers. This local IgA response was significant given the observation that protective immunity against non-invasive, gut-associated bacterial pathogens such as ETEC occurs at the mucosal surface of the small intestine (Levine et al., 1979). Although the conjugate was administered by a parenteral route (s.c.), numerous studies indicate that systemic administration of polysaccharide-based conjugate vaccines can induce immune responses at the local mucosal level and, perhaps as a result, also reduce nasopharyngeal carriage (Zhang and Finn, 2004). In line with this latter notion, mice vaccinated with the PD-O148 conjugate were protected against ETEC infection as evidenced by the significantly decreased ETEC stool shedding, which has been shown to correlate with a reduction in the presence of diarrhea (Lindsay et al., 2014). We suspect that the reduced ETEC colonization observed here resulted from the ability of the PD-O148 conjugate to trigger both systemic and mucosal immune responses, although further investigation of the mechanisms of immunity induced by the conjugate, especially as a function of immunization route, are clearly needed.

Our findings expand the repertoire of available ETEC antigens to include molecules that have largely been overlooked for vaccine development and support the further investigation of ETEC O-PS as a subunit vaccine antigen. This antigenic expansion is significant for several reasons. First, even though glycoconjugates have a track record of safely and effectively preventing bacterial infections, there are surprisingly few that have been fully licensed. That said, these numbers are poised to increase in the coming years as cell-based and cell-free technologies for glycoengineering recombinant vaccines, such as those described here and elsewhere (Kay et al., 2019), reach full maturity. As part of this maturation, it is imperative to continue growing the pipeline with as many promising anti-bacterial vaccine candidates as possible, especially as diarrheal and other vaccine-preventable diseases continue to be unmet challenges and as antibiotic-resistant bacteria could threaten as many as 10 million people annually by 2050 (O’Neill, 2014). Second, all ETEC vaccines that have entered clinical trials to date are focused on the classical paradigm of ETEC pathogenesis and seek to induce immune responses to one or more CF/CS antigens and LT (Bourgeois et al., 2016; Riddle et al., 2018; Fleckenstein, 2021; Khalil et al., 2021). However, the risk of focusing on the classical paradigm is that it limits vaccine development to a subset of canonical antigens, which could be problematic if our current view of ETEC pathogenesis is incomplete, as has been suggested recently (Fleckenstein, 2021). For this reason, we decided to investigate O-PS from ETEC serogroups O148 and O78 with the goal of validating these non-canonical targets and supplementing the existing inventory of ETEC vaccine antigens. One potential drawback of O-PS as an ETEC vaccine target is the fact there are more than 78 O serogroups. Fortuitously, however, as few as 10 serogroups (O6, O8, O9, O25, O27, O78, O128, O148, O153 and O159) account for >75% of known ETEC isolates (Wolf, 1997), suggesting that a 10-valent conjugate vaccine could afford broad protection. This number is much more feasible for multivalent ETEC vaccine development, especially considering that some of the most effective licensed conjugates achieve a valency of >10 by attachment of distinct polysaccharides from the most important serogroups (*e.g.*, Prevnar13, Prevnar20). It is also worth noting that multivalency is not a unique challenge for an O-PS-based vaccine. There are 25 distinct CF/CS antigens identified to date and a subset of these would need to be combined in some manner in order to achieve ∼75% coverage of all isolates expressing the most common colonization factors (Fleckenstein, 2021). Hence, both O-PS and protein antigen-based vaccine candidates face complicated paths to a broadly protective, multivalent vaccine.

The ETEC O148 and O78 structures are now part of an ever-expanding list of polysaccharides that can be transferred to acceptor proteins by the *Cj*PglB biocatalyst. *Cj*PglB is well known for its remarkably relaxed oligosaccharide substrate specificity that allows transfer of diverse Und-PP-linked glycans including numerous different O-PS structures (Feldman et al., 2005; Wacker et al., 2006). The ability of *Cj*PglB to site-specifically modify diverse acceptor proteins is aided by the introduction of a genetically encoded *N-*linked glycosylation tag that can be appended N- or C-terminally in single or multiple copies, or can be inserted at internal locations in the acceptor protein (Fisher et al., 2011). Here, the introduction of four tandemly repeated DQNAT motifs at the C-termini of CRM_197_ and PD facilitated their use as acceptor protein substrates for *Cj*PglB and significantly expanded the set of carrier proteins available for bioconjugation, which historically has focused on a narrow set of carriers that are not currently used in any licensed vaccines—most notably *Pseudomonas aeruginosa* exotoxin A (ExoA) (Kay et al., 2019). Importantly, the in-built flexibility of bacterial OSTs like *Cj*PglB together with programmable glycosylation motifs makes it straightforward to produce an array of custom vaccines comprised of different polysaccharide/protein combinations.

According to the Centers for Disease Control and Prevention (CDC), the cost of conjugate vaccines ranges between about $10 per dose (e.g*.*, ActHIB for *Haemophilus influenzae*) up to ∼$75–125 per dose (e.g., Menactra for meningococcal disease; Prevnar 13 for pneumococcal disease) (CDC, 2019). The cell-based and cell-free strategies described here represent greatly simplified alternatives for biomanufacturing conjugate vaccines whereby metabolically engineered *E. coli* or their cell-free extracts are exploited for one-step production of an unlimited and renewable supply of pure conjugate vaccine product. Because of the dramatic simplification of the process, bioconjugation strategies are anticipated to allow scalable production of large quantities of conjugates at a much more affordable cost, which is especially important for achieving sustained impact on global public health. We anticipate that a large-scale, *E. coli*-based manufacturing process for conjugates would offer a highly competitive cost per dose as was described recently for *E. coli*-based production of a biologic antiviral (Decker et al., 2020). Likewise, our own economic analysis of cell-free production revealed that iVAX reactions are inexpensive (Stark et al., 2021) and costs can be further decreased by optimizing the cell-free extract formulation to use the low-cost energy substrate maltodextrin in place of the significantly more expensive phosphorylated secondary energy substrate phosphoenolpyruvate (PEP) (Warfel, 2022). Additional advantages of cell-free systems for conjugate production include that they can be: i) distributed through freeze drying (Hunt et al., 2017) and rehydrated at the point of use by a “just add water” approach (Pardee et al., 2016; Stark et al., 2021); ii) linearly scaled from 1 nL to 100 L (Zawada et al., 2011) for accelerated process development; and iii) rapidly customized and reconfigured for product switching (Pardee et al., 2016; Adiga et al., 2018). Collectively, the cell-based and cell-free platforms described here lay the foundation for future creation of a custom, multivalent vaccine with the potential for broad ETEC coverage and increased access through adoption of simplified, low-cost biomanufacturing platforms.

## Materials and methods

### Bacterial strains and plasmids

All strains used in this study are provided in Supplementary Table S1. Briefly, *E. coli* strain DH5α was used for all molecular biology including plasmid cloning and isolation. *E. coli* W3110 was used for expressing O-PS on lipid A-core and displaying O-PS molecules on the cell surface while *E. coli* CLM24 (Feldman et al., 2005) was used as the host strain for expressing glycoengineered conjugates using intact cells. CLM24 is a derivative of W3110 that carries a deletion in the gene encoding the WaaL ligase, thus facilitating the accumulation of preassembled glycans on Und-PP as substrates for *Cj*PglB-mediated protein glycosylation. CLM24 Δ*lpxM* was used as the source strain for expressing glycoengineered conjugates in cell-free reactions. CLM24 Δ*lpxM* lacks the gene encoding the lipid A acyltransferase LpxM, a deletion that yields a pentaacylated lipid A structure with significantly reduced toxicity (Needham et al., 2013). The ETEC strains B7A (serotype O148:H28; CS6, LT, STa) (DuPont et al., 1971) and H10407 (serotype O78:H11; CFA/I, LT, STa) (Levine et al., 1980) were used for SBA and challenge studies as well as a source of LPS. These strains were chosen because both were previously used in human challenge trials (Levine et al., 1979; Levine et al., 1980).

Plasmids used in the study are listed in Supplementary Table S1. Briefly, plasmids pTrc99A-ssDsbA-PD^4xDQNAT^ and pTrc99A-ssDsbA-CRM_197_^4xDQNAT^ (Stark et al., 2021) were used to express the PD and CRM_197_ acceptor proteins in the *E. coli* periplasm. These plasmids were constructed by PCR amplification of the ssDsbA-PD^4xDQNAT^ and ssDsbA-CRM_197_^4xDQNAT^ sequences from plasmids pTrc99S-ssDsbA-PD^4xDQNAT^ and pTrc99S-ssDsbA-CRM_197_^4xDQNAT^, respectively (Stark et al., 2021), and followed by ligation of the PCR products in pTrc99A. Plasmid sequences were confirmed by Sanger sequencing at the Genomics Facility of the Cornell Biotechnology Resource Center (BRC). Plasmids pMAF10 (Feldman et al., 2005) and pMAF10^D54N/E316Q^ (Ollis et al., 2014) were used for cell-based expression of wild-type CjPglB and an inactive D54N/E316Q mutant of *Cj*PglB, respectively. The bacterial O-PS biosynthetic pathway plasmids were pMW07-O78 (Chen et al., 2016; Stark et al., 2021; Warfel, 2022) for expressing the O-PS of ETEC strain H10407 and pMW07-O148 (Celik et al., 2015; Chen et al., 2016) for expressing the O-PS of ETEC strain B7A. Plasmids used for cell-free expression of conjugate vaccines included pSF-*Cj*PglB-LpxE for co-expressing CjPglB along with *Francisella tularensis* phosphatase LpxE that promotes monophosphorylation of lipid A (Stark et al., 2021) and pJL1-PD^4xDQNAT^ for expressing PD^4xDQNAT^ (Stark et al., 2021).

### Cell-based glycoconjugate expression and purification

For cell-based glycoconjugate expression, plasmids pTrc99A-ssDsbA-PD^4xDQNAT^ and pTrc99A-ssDsbA-CRM_197_^4xDQNAT^ encoding conjugate carrier proteins preceded by the DsbA signal peptide for translocation to the periplasm were used to transform CLM24 cells carrying a bacterial O-PS pathway encoded on plasmid pMW07-O148 or pMW07-O78 and *Cj*PglB encoded on plasmid pMAF10 or pMAF10^D54N/E316Q^. Transformed cells were grown in 10 mL LB medium (10 g/L yeast extract, 5 g/L tryptone, 10 g/L NaCl) overnight at 37°C. The next day, cells were subcultured into 1 L of LB and allowed to grow at 37°C until the optical density at 600 nm (OD_600_) reached 0.6–0.8. The culture was then supplemented with 0.2% arabinose to induce expression of *Cj*PglB and grown at 30°C for 16 h, after which 0.5 mM isopropyl-β-D-thiogalactopyranoside (IPTG) was added to induce expression of the conjugate carrier protein for an additional 8 h at 30°C. The cells were harvested, and cell pellets were resuspended in lysis buffer (20 mM Tris-HCl, 200 mM NaCl, 10 mM imidazole; pH 7.5) at 2–5 mL buffer per Gram wet weight. Cells were lysed using a EmulsiFlex-C5 homogenizer (Avestin) then centrifuged at 13,000 x g for 30 min. The lysate was filtered through a 0.45-μm syringe filter and loaded onto a gravity flow column containing Ni-NTA resin (Thermo Fisher Scientific) that was washed with 5–10 column volumes of wash buffer (20 mM Tris-HCl, 200 mM NaCl, 20 mM imidazole; pH 7.5). The resin and cell lysate supernatant were allowed to equilibrate for 30 min at 4°C. After flowthrough of the supernatant, the resin was washed with five column volumes of wash buffer. The protein was eluted with three column volumes of elution buffer (20 mM Tris-HCl, 200 mM NaCl, 300 mM imidazole; pH 7.5). Eluted protein was dialyzed into sterile PBS, concentrated, and quantified measured by Bradford assay.

### Periplasmic extract preparation

Periplasmic extracts containing the expressed glycoproteins were prepared by centrifuging the induced cultures at 13,000 x g and 4°C for 2 min. The resulting pellets were resuspended in 0.4 M L-arginine (Sigma-Aldrich; 100 μL 0.4 M L-arginine per 100 mL culture) and incubated at 4°C for 1 h with gentle shaking at 10-min intervals. The resuspended pellets were then centrifuged as above to obtain the final periplasmic extracts in the supernatant.

### Cell-free extract preparation

Cell-free extracts were prepared as previously described (Jaroentomeechai et al., 2018; Warfel, 2022). Specifically, CLM24 Δ*IpxM* cells were transformed with both pSF-*Cj*PglB-LpxE and pMW07-O78 plasmids for the strain used to generate PD-O78, and only the pSF-*Cj*PglB-LpxE plasmid for the strain used to generate the negative control aglycosylated PD. Cells were grown in a Sartorius Stedim BIOSTAT Cplus bioreactor at the 10-L scale in 2xYTP media supplemented with carbenicillin at 100 μg/mL and chloramphenicol at 34 μg/ml or only carbenicillin at 100 μg/mL in the negative control extract. Cells were inoculated at OD_600_ ≈ 0.08 and induced at OD_600_ ≈ 1 with 0.02% arabinose to induce expression of *Cj*PglB and O-PS enzymes and harvested at OD_600_ ≈ 3. All subsequent steps were performed on ice unless otherwise stated. Cells were harvested by centrifugation at 5,000 x g for 15 min and then washed 3 times with S30 buffer (10 mM Tris acetate pH 8.2, 14 mM magnesium acetate, and 60 mM potassium acetate). Following washing, cells were pelleted at 7,000 x g for 10 min, then flash frozen and stored at −80°C. For lysis, CLM24 Δ*lpxM* cells were resuspended in 1 mL/g S30 buffer then homogenized using an EmulsiFlex-C3 high-pressure homogenizer (Avestin) with 1 pass at a pressure of ∼21,000 psig. Following lysis, cells were centrifuged for 12,000 *g* for 10 min. Supernatant was then collected and incubated at 37°C for 1 h in a runoff reaction. Cells were then centrifuged once more at 10,000 x g for 10 min and the supernatant was flash frozen and stored at −80°C as the final extract.

### Cell-free protein synthesis reactions

For cell-free synthesis of PD-O78 and aglycosylated PD, reactions were prepared at the 5-mL scale in 50 mL conical tubes. Reactions producing PD-O78 used extract enriched with both *Cj*PglB and ETEC-O78 O-PS, while reactions producing aglycosylated PD used extract enriched only with PglB. Each reaction was prepared as described previously (Hershewe et al., 2021) to contain 3.33 ng/μL pJL1-PD-4xDQNAT plasmid and 30% (vol./vol%) extract in addition to: 10 mM magnesium glutamate (Sigma, 49605), 10 mM ammonium glutamate (Biosynth, FG28929), 130 mM potassium glutamate (Sigma, G1501), 1.2 mM adenosine triphosphate (Sigma A2383), 0.85 mM guanosine triphosphate (Sigma, G8877), 0.85 mM uridine triphosphate (Sigma U6625), 0.85 mM cytidine triphosphate (Sigma, C1506), 0.034 mg/mL folinic acid, 0.171 mg/mL *E coli* tRNA (Roche 10108294001), 2 mM each of 20 amino acids, 30 mM phosphoenolpyruvate (PEP, Roche 10108294001), 0.4 mM nicotinamide adenine dinucleotide (Sigma N8535-15VL), 0.27 mM coenzyme-A (Sigma C3144), 4 mM oxalic acid (Sigma, PO963), 1 mM putrescine (Sigma, P5780), 1.5 mM spermidine (Sigma, S2626), 57 mM HEPES (Sigma, H3375), and 15–20 μg/mL T7. Reactions were then lyophilized for 16–20 h using a VirTis Benchtop Pro Lyophilizer (SP scientific). Fully lyophilized reactions were rehydrated with 5 mL nuclease-free water and incubated at 30°C for 1 h to synthesize the carrier protein (PD). After 1 h of protein synthesis, glycosylation was initiated by supplementing 25 mM MnCl_2_ and 0.1 % wt/vol DDM. Reactions were incubated for one more hour at 30°C, then were centrifuged at 16,000 x g for 15 min. The His-tagged carrier protein was then purified from the soluble cell-free reactions using Ni-NTA affinity resin as previously described (Warfel, 2022).

### Western blot analysis

Cell-based glycoconjugate samples were run on NuPAGE 4%–12% Bis-Tris gels (Invitrogen). Following electrophoretic separation, proteins were transferred from gels onto 0.45-μm Immobilon-P polyvinylidene difluoride membranes (PVDF) using a mini blot module (Thermo Fisher Scientific) according to the manufacturer’s instructions. Membranes were washed twice with TBS buffer (80 g/L NaCl, 20 g/L KCl, and 30 g/L Tris-base) followed by incubation for 1 h in blocking solution (50 g/L non-fat milk in TBS). After blocking, membranes were washed three times in TBS-T (TBS with 0.05% (v/v%) Tween-20) with a 5-min incubation between each wash. For fluorescence-based detection of immunoblots, membranes were probed with both an anti–6x-His tag antibody (R&D Systems, Cat # MAB050; diluted 1:7,500) and anti–ETEC O148 antibody (Abcam, Cat # ab78827; diluted 1:1,000) or anti–ETEC O78 antibody (Abcam, Cat # ab78826; diluted 1:1,000) in 1X TBS-T with 5% (w/v) BSA. Probing of membranes was performed overnight at 4°C with gentle rocking, after which membranes were washed with TBS-T as described above and probed with fluorescently labeled secondary antibodies for 1 h at room temperature in 1X TBS-T with 5% (w/v) nonfat dry milk. The membrane was washed for 5 min with TBS-T and then imaged using a ChemiDoc XRS + System (Bio-Rad). For chemiluminescence-based detection of immunoblots, membranes were probed with an anti–6x-His tag antibody (Abcam, Cat # ab9108; diluted 1:7,500) and then with the corresponding anti-mouse HRP-conjugated secondary antibody (Abcam, Cat # ab205718; diluted 1:7,500). Another membrane was separately probed with anti-ETEC O148 antibody (Abcam, Cat # ab78827; diluted 1:1,000) or anti-ETEC O78 antibody (Abcam, Cat # ab78826; diluted 1:1,000) in 1X TBS-T with 5% (w/v) BSA and then with anti-rabbit HRP-conjugated secondary antibody (Abcam, Cat # ab205718; diluted 1:7,500). For signal visualization, membranes were briefly incubated at room temperature with Western ECL substrate (Bio-Rad) and imaged using a ChemiDoc XRS + System (Bio-Rad).

Cell-free samples were run on 4%–12% Bis-Tris gels with SDS-MOPS running buffer supplemented with NuPAGE antioxidant. Samples were then transferred to PVDF 0.45-μm membranes (Millipore, USA) for 55 min at 80 mA per blot using a semi-dry transfer cell. Membranes were blocked for 1 h at room temperature or overnight at 4°C in Intercept Blocking Buffer (Licor). Primary antibodies used were anti-His antibody (Abcam, Cat # ab1187; diluted 1:7,500) or anti-ETEC-O78 antibody (Abcam, Cat # ab78826; diluted 1:2,500) in Intercept blocking buffer with 0.2% (v/v) Tween 20, and membranes were incubated for 1 h at room temp or overnight at 4°C. The secondary antibody used was a fluorescent goat anti-rabbit antibody (Licor, Cat # GAR-680RD; diluted 1:10,000) in Intercept blocking buffer, 0.2% (v/v) Tween 20% and 0.1% (w/v) SDS for both anti-His and anti-ETEC-O78 blots. Blots were washed 6 times for 5 min after each of the blocking, primary, and secondary antibody incubations using PBS-T. Blots were imaged with Licor Image studio.

### Dot blot analysis

To detect cell-surface expression of ETEC O148 O-PS, overnight cultures of the following strains were grown: *E. coli* strains W3110 and CLM24 without a plasmid, W3110 carrying empty pMW07, W3110 and CLM24 carrying pMW07-O148, and ETEC strain B7A. A total of 2 μL containing an equivalent amount of each strain, as well as LPS extracted from B7A cells, were spotted onto a nitrocellulose membrane. The membrane was allowed to dry, and then non-specific sites were blocked by soaking in 5% (w/v) BSA in TBS-T for 1 h at room temperature, followed by incubation for 30 min with anti–ETEC O148 antibody (Abcam, Cat # ab78827; diluted 1:1,000) in 0.1% (w/v) BSA in TBS-T. The membrane was washed three times with TBS-T, then incubated with secondary antibody conjugated to HRP. After three TBS-T washes, the membrane was incubated with Western ECL substrate (Bio-Rad) and imaged using a ChemiDocTM XRS + System (Bio-Rad). An identical protocol was followed for detection of cell-surface expression of ETEC O78 O-PS.

### Immunization

Groups of eight 6-week-old female BALB/c mice (Harlan Sprague Dawley) were immunized s. c. with 50 μL of sterile PBS (pH 7.4, Fisher Scientific) or formulations containing either aglycosylated PD or PD-O148 conjugate. The amount of antigen in each preparation was normalized such that ∼25–50 μg of these proteins was administered per injection. The purified protein groups were formulated in sterile PBS and mixed with an equal volume of Adju-Phos aluminium phosphate adjuvant (InvivoGen) before injection. Mice were boosted 21 and 42 days after the initial immunization. For antibody titering, blood was taken on days 0, 35, and 49 *via* submandibular collection, as well as at the study termination on day 56 *via* cardiac puncture. Sera were isolated from the collected blood draws after centrifugation at 5,000 x g for 10 min and stored at −20°C. For bacterial killing assays, final blood serum collections for all the mice within each group were pooled. The protocol number for the animal trial was 2012–0132 and was approved by the Institutional Animal Care and Use Committee (IACUC) at Cornell University.

### Serum antibody titering

Serum IgG antibody titers were determined by ELISA using LPS derived from ETEC strains as immobilized antigen. Specifically, O148 and O78 LPS molecules were prepared from ETEC strains B7A and H10407, respectively, by hot phenol water extraction and DNase I (Sigma) and proteinase K (Invitrogen) treatment, as described elsewhere (Svennerholm et al., 2022). Briefly, extracted LPS samples were purified using PD-10 desalting columns packed with Sephadex G-25 resin (Cytiva), and concentrations were determined using a purpald assay (Lee and Tsai, 1999). 96-well plates (MaxiSorp; Nunc Nalgene) were incubated with 0.5 μg/mL of purified LPS diluted in PBS, pH 7.4, 25 μL/well, at 4°C overnight. Plates were incubated with 50 μL blocking buffer (5% (w/v) nonfat dry milk (Carnation) in PBS) overnight at 4°C, then washed three times with 200 μL PBS-T (PBS, 0.05% (v/v) Tween 20) per well. Serum samples isolated from the collected blood draws of immunized mice were appropriately serially diluted in triplicate in blocking buffer and added to the plates for 2 h at 37°C. Plates were washed three times with PBS-T (+0.03% BSA (w/v)), then incubated for 1 h at 37°C in the presence of a horseradish peroxidase–conjugated goat anti-mouse IgG antibody (Abcam, Cat # ab97265; diluted 1:25,000). After three PBS-T + 0.3% BSA washes, 50 μL of 3,3′-5,5′-tetramethylbenzidine substrate (1-Step Ultra TMB-ELISA; Thermo Fisher Scientific) was added to each well, and the plates were incubated at room temperature in the dark for 30 min. The reaction was stopped by adding 50 μL of 2 M H_2_SO_4_, and absorbance was measured at a wavelength of 450 nm using a FilterMax F5 microplate spectrophotometer (Agilent). Serum antibody titers were determined by measuring the lowest dilution that resulted in signals that were 3 standard deviations above the background controls of no serum.

### Fecal IgA quantification

Fecal samples collected from immunized mice were fully resuspended in sterile PBS at a concentration of ∼50 mg/mL by weight. The solids were separated by centrifuging for 5 min at 16,000 x g and 4°C, and supernatants were stored at −20°C until use. For O148 O-PS-specific IgA detection, 96-well ELISA plates (MaxiSorp; Nunc Nalgene) were coated with 0.5 μg/mL of purified O148 LPS diluted in PBS, pH 7.4, 25 μL/well, and incubated at 4°C overnight. Plates were next incubated with 50 μL blocking buffer (5% (w/v) nonfat dry milk in PBS) overnight at 4°C, then washed three times with PBS-T. Appropriately diluted fecal samples were added and incubated at 37°C for 2 h. After incubation, plates were washed three times with PBS-T (supplemented with 0.03% BSA (w/v)), then incubated for 1 h at 37°C in the presence of HRP-conjugated goat anti-mouse IgA antibody (Fortis Life Biosciences, Cat # A90-103P Lot #56; diluted 1:10,000). After three PBS-T + 0.3% (w/v) BSA washes, ELISA plates were developed by 1-Step Ultra TMB-ELISA (Thermo Fisher Scientific) and quenched by 2 M H_2_SO_4_. The colorimetric reactions were assayed at a wavelength of 450 nm using a FilterMax F5 microplate spectrophotometer (Agilent).

### SBA

A modified version of a previously described SBA method was followed (Valentine et al., 2016). ETEC B7A cells were grown overnight from a frozen glycerol stock, then seeded 1:20 in LB medium. Log-phase grown bacteria were harvested, adjusted to an OD_600_ of 0.1, then further diluted 1:5,000 in Hanks’ Balanced Salt Solution with 0.5% (w/v) BSA (Sigma Aldrich). Assay mixtures were prepared in 96-well microtiter plates by combining 20 μL of serially diluted heat-inactivated test serum (dilutions ranging from 10^0^–10^4^), and 10 μL of diluted bacterial suspension. After incubation with shaking for 60 min at 37°C, 10 μL of active or inactive complement source was added to each well, to a final volume percent of 25% (v/v). Heat-inactivated complement was prepared by thawing an aliquot of active pooled human complement serum (Innovative Research, ICSER1ML), incubating in a 56°C water bath for 30 min, and cooling at room temperature. Assay plates were incubated with shaking at 37°C for 60–90 min, then 10 μL was plated from each well (diluted to 50 μL in LB) on LB agar plates. Serum samples were tested and plated in duplicate, and colonies were counted (Promega Colony Counter) after 16–18 h of incubation at 30°C. CFUs were counted for each individual serum dilution, and SBA titers were determined by calculating percent survival at various serum dilutions. Data was plotted as percentage survival *versus* serum dilution.

### ETEC infection

Groups of eight 6-week-old female BALB/c mice (Harlan Sprague Dawley) were immunized s. c. with 50 μL of sterile PBS (pH 7.4, Fisher Scientific) or formulations containing aglycosylated PD or PD-O148 conjugate, according to the 49-day immunization schedule described above. At 7 days post-vaccination (48 h prior to challenge infection), mice received streptomycin (5 g/L) and fructose (6.7% (w/v)) in the drinking water to eradicate normal flora and fecal pellets were collected. Food was withheld 12 h prior to challenge infection and replaced with sterile water without antibiotics. Famotidine (50 mg/kg) (Sigma-Aldrich) was then administered 2 h prior to challenge infection to neutralize gastric acid. At 9 days post-vaccination, mice were subsequently infected with a 200-μL inoculum containing ∼1 × 10^4^ CFU of ETEC strain B7A, administered by gavage with a feeding needle directly introduced in the stomach *via* the esophagus. After challenge infection, mice were checked daily for 3 days, and fecal pellets were collected and stored at −20°C for further analysis. Mice were sacrificed 72 h post-infection. All procedures were carried out in accordance with protocol 2012-0132 approved by the Cornell University Institutional Animal Care and Use Committee.

### Quantitative real-time PCR analysis of ETEC burden

DNA from fecal pellets of individual mice was extracted from thawed stool samples using a QIAamp DNA stool kit (Qiagen) following the manufacturer’s instructions. To enhance extraction of pathogen DNA, stool samples were first vigorously homogenized with ∼300 mg of 1.0-mm-diameter zirconia beads (Bio Spec) using a Mini-Bead Beater (BioSpec). After extraction, DNA was eluted in elution buffer and stored at −20°C. Stool DNA and tissue were analyzed for the ETEC B7A-specific heat-labile enterotoxin LT encoded by the *eltA* gene to determine the levels of shedding of the organism in stool. Quantification of ETEC was performed by qPCR using Taq DNA polymerase, as described elsewhere (Panchalingam et al., 2012; Liu et al., 2013), using the following conditions: preheating at 95°C for 5 min, denaturation at 95°C for 30 s, annealing at 58°C for 30 s, elongation at 72°C for 1 min. PCR was performed for 55–60 cycles for maximal saturation of signal with final extension at 72°C for 7 min in a 7500 Fast Real-Time PCR System (Applied Biosystems). The primer sequences used were: *eltA* forward 5′- TTCCCACCGGATCACCAA -3′ and *eltA* reverse 5′-CAA​CCT​TGT​GGT​GCA​TGA​TGA - 3′, along with a custom Taqman Probe (Thermo Fisher Scientific) 5′- CTT​GGA​GAG​AAG​AAC​CCT-3′ labeled with FAM (6-carboxyfluorescein) at the 5′ end and MGB at the 3′end. Reactions with no DNA template were also included as controls. Reaction components were combined in MicroAmp 96-well reaction plates (Applied Biosystems) and plates were centrifuged at ∼500 x g for 1 min before each reaction.

### Statistical analysis and reproducibility

To ensure robust reproducibility of all results, experiments were performed with at least three biological replicates and at least three technical measurements. Sample sizes were not predetermined based on statistical methods but were chosen according to the standards of the field (at least three independent biological replicates for each condition), which gave sufficient statistics for the effect sizes of interest. All data were reported as average values with error bars representing standard error of the mean (SEM). Data were analyzed for statistical significance using the Mann-Whitney test using GraphPad Prism 9 for MacOS (Version 9.4.1). All graphs were also generated using Prism 9 for MacOS. No data were excluded from the analyses. The experiments were not randomized. The Investigators were not blinded to allocation during experiments and outcome assessment.

## Data Availability

The original contributions presented in the study are included in the article/Supplementary Material, further inquiries can be directed to the corresponding author.
